# Long‐term patient‐reported outcomes of open urorectal fistula repair after prostate cancer treatment

**DOI:** 10.1111/bju.70233

**Published:** 2026-03-13

**Authors:** Max C. Wagner, Jakob Klemm, Navid Roessler, Robert J. Schulz, Dejan K. Filipas, Margit Fisch, Roland Dahlem, Malte W. Vetterlein

**Affiliations:** ^1^ Department of Urology University Medical Center Hamburg‐Eppendorf Hamburg Germany

**Keywords:** patient‐reported outcome measures, postoperative complications, prostatectomy, surgical flaps, urorectal fistula

## Abstract

**Objectives:**

To evaluate long‐term outcomes of open urorectal fistula (URF) repair, including URF recurrence, need for re‐intervention, and patient‐reported outcomes.

**Patients and Methods:**

This retrospective study included men undergoing open URF repair between 2014 and 2024. Data collected encompassed comorbidities, prostate cancer treatment history, prior URF interventions, and intraoperative details. Endpoints were: (i) URF recurrence‐free survival, (ii) re‐intervention‐free survival (no further disease‐related procedures), and (iii) validated patient‐reported outcome measures (PROMs). Kaplan–Meier estimators were used for survival analyses; PROMs were scored according to standard protocols.

**Results:**

A total of 29 patients underwent open URF repair. The median (interquartile range [IQR]) age was 68 (61–71) years, body mass index was 26 (23–28) kg/m^2^, and the time from prostatectomy to URF repair was 10 (4–13) months. Five patients (17%) had prior pelvic radiotherapy; 13 (45%) underwent redo repairs. Presenting symptoms included rectal urine leakage (48%), pneumaturia (24%), recurrent infections (21%), dysuria (21%), and faecaluria (10%). Transperineal repair was performed in 26 patients (90%) and transabdominal repair in three (10%). The median (IQR) operating time was 90 (80–107) min. The median follow‐up was 50 months for recurrence and 58 months for re‐intervention. The 5‐year URF recurrence‐free and any disease‐related re‐intervention‐free survival estimates were 96% and 75%, respectively. The median (IQR) six‐item lower urinary tract symptoms score from the Urethral Stricture Surgery PROM was 4 (2–8), International Consultation on Incontinence Questionnaire–Urinary Incontinence Short Form sum score was 11 (6–15), Wexner faecal incontinence score was 3 (1–9), International Consultation on Incontinence Questionnaire–Satisfaction outcome score was 21 (18–23), and Decision Regret Scale score was 0 (0–10), indicating restored voiding function, moderate urinary incontinence, mild faecal incontinence, high patient satisfaction, and negligible decisional regret.

**Conclusion:**

Open URF repair achieves durable URF closure with favourable long‐term outcomes, even in complex cases. Patient satisfaction is high, while moderate urinary incontinence persists in some, likely reflecting underlying disease. Voiding and faecal continence remain largely preserved.

**Abbreviations**
BMI: body mass index
ICIQ‐S: ICIQ‐Satisfaction
ICIQ‐UI SF: International Consultation on Incontinence Questionnaire–Urinary Incontinence Short Form
PROM: patient‐reported outcome measure
IQR: interquartile range
URF: urorectal fistula
USS PROM: Urethral Stricture Surgery Patient‐Reported Outcome Measure

## Introduction

Urorectal fistulas (URFs) are a rare but serious complication following treatment for prostate cancer and are associated with substantial impairment in quality of life. The incidence of rectal injury during radical prostatectomy is ~0.58%, although this varies with surgical approach and patient‐specific risk factors [1]. Given the high global volume of prostatectomies, even a low incidence results in a meaningful number of affected patients, rendering URFs a clinically relevant and distressing complication for patients and clinicians alike.

Major complications after oncological surgery, such as URFs following prostatectomy, impose both psychological and physical burdens. Beyond the initial stress of cancer diagnosis and treatment, patients who develop a URF may experience ongoing distress and markedly reduced quality of life. As long‐term survival following prostate cancer treatment is generally favourable, preservation of quality of life becomes increasingly important. Currently, no disease‐specific guidelines from major urological or colorectal societies address the management of URFs following local therapy for prostate cancer. Existing literature—including systematic reviews [2, 3] and large multi‐institutional series [4, 5, 6]—consistently emphasises the lack of standardised, evidence‐based protocols, particularly in radiation‐associated or complex cases.

Despite the clinical relevance of URFs, data on patient‐centred outcomes following surgical repair remain limited. To address this gap, the aim of the present study was to evaluate long‐term outcomes after URF repair, with particular emphasis on re‐treatment rates and patient‐reported outcomes, including urinary and faecal continence, voiding symptoms, overall treatment satisfaction, and decisional regret.

## Patients and Methods

### Study Population and Data Extraction

This retrospective observational study was conducted in accordance with local regulations (Hamburg Hospital Act, HmbKHG §12.1) and was approved by the Ethics Committee of the Medical Council of Hamburg (Institutional Review Board number: 2021‐100 628‐BO‐ff). Men who underwent URF repair between January 2014 and June 2024 were identified using the operation and procedure code (OPS) 5–578.30. Patients who underwent URF repair for non‐prostate cancer aetiologies were excluded. Clinical and surgical data were extracted following review of electronic medical records. Major postoperative complications within 30 days (Clavien–Dindo Grade ≥III) were recorded.

### Follow‐Up and Definition of Outcomes

Cross‐sectional follow‐up was performed via telephone interview and an online survey. Objective outcomes were defined as: (i) URF closure, confirmed by voiding cystourethrography at 21 days and absence of URF re‐treatment at last follow‐up; and (ii) freedom from re‐intervention for any disease‐related events.

Subjective outcomes were assessed using validated German‐language patient‐reported outcome measures (PROMs): the six‐item LUTS score from the Urethral Stricture Surgery (USS) PROM [7], the International Consultation on Incontinence Questionnaire–Urinary Incontinence Short Form (ICIQ‐UI SF) [8], and the Wexner faecal incontinence score [9]. Treatment satisfaction was evaluated using the ICIQ‐Satisfaction (ICIQ‐S) [10], which yields both an outcome score and a global satisfaction item. Decisional regret was measured using the five‐item Decision Regret Scale (DRS) [11].

### Surgical Procedure and Perioperative Management

Preoperative evaluation was conducted according to institutional protocol and included clinical assessment, urine analysis, cystoscopy, and combined retrograde and voiding cystourethrography. All surgeries were performed by one of two high‐volume reconstructive urologists (M.F., R.D.). A transperineal or transabdominal approach was selected based on patient history, URF location and extent, radiation history, and surgeon preference.

Our surgical technique has been described previously [12]. In brief, the transabdominal procedure involves complete excision of the URF, followed by multilayer closure of the bladder and urethra. An omental flap is interposed between the suture lines to promote healing and minimise the risk of recurrence. A Foley catheter and suprapubic tube are placed to ensure adequate urinary drainage, and the bladder is closed in two layers. For the transperineal approach, the patient is positioned in an exaggerated lithotomy position to allow optimal access to the perineum. The fistula tract is excised via a perineal incision, and a pedicled ischiorectal fat flap is mobilised and interposed between the urethra and bladder to reinforce the repair. As with the transabdominal technique, both a Foley catheter and suprapubic tube are inserted to provide urinary diversion during healing. A standardised radiographic voiding trial, including voiding cystourethrography, is performed at 3 weeks postoperatively to confirm URF closure and assess bladder emptying.

### Statistical Analyses

Baseline characteristics were summarised using descriptive statistics. Continuous variables were reported as medians with interquartile ranges (IQRs) and categorical variables as counts and percentages. Median follow‐up for censored observations was calculated using the reverse Kaplan–Meier method. Patients without a documented recurrence or re‐intervention and a follow‐up of <3 months were considered lost to follow‐up. URF recurrence‐free and re‐intervention‐free survival were analysed using Kaplan–Meier estimation, with patients censored at the time of last known follow‐up or time of death.

The PROMs were evaluated according to standard scoring instructions. LUTS scores range from 0 to 24 [7], ICIQ‐UI SF from 0 to 21 [8], and Wexner faecal incontinence scores from 0 to 20 [9], with higher scores reflecting greater symptom burden. The ICIQ‐S outcome score ranges from 0 to 24, and the global satisfaction item from 0 to 10 [10], with higher values indicating greater satisfaction. DRS scores range from 0 to 100 [11], with higher scores reflecting greater regret. Results were summarised as median (IQR), and distributions were visualised using violin plots. To account for non‐response bias, clinical characteristics of patients with PROMs responders were compared to PROMs non‐responders using the Mann–Whitney *U* test, chi‐squared test, or Fisher's exact test, as appropriate.

All statistical analyses were performed using Stata® (StataCorp. 2023. Stata Statistical Software: Release 18. StataCorp LLC, College Station, TX, USA).

Assistance in language editing was provided by Chat Generative Pre‐trained Transformer (ChatGPT)‐5 (OpenAI, Inc., San Francisco, CA, USA). The tool was used exclusively for linguistic editing; no content, interpretations, or conclusions were produced by artificial intelligence.

### Scoping Review on Outcomes of URF Repair After Radical Prostatectomy

To assess clinical outcomes and the use of validated PROMs in the literature on URF repair, we conducted a comprehensive scoping review on 14 January 2025. The search was performed in PubMed using the following search terms: ‘prostate cancer’ AND (‘recto‐vesical fistula’ OR ‘rectovesical fistula’ OR ‘vesicorectal fistula’ OR ‘vesico‐rectal fistula’ OR ‘uro‐rectal fistula’ OR ‘urorectal fistula’ OR ‘recto‐urinary fistula’ OR ‘rectourinary fistula’ OR ‘rectourethral fistula’ OR ‘recto‐urethral fistula’ OR ‘urethrorectal fistula’ OR ‘urethro‐rectal fistula’). Studies were excluded if they included <10 patients, were non‐English, or addressed fistulas unrelated to prostate cancer therapy. Reference lists of eligible articles were hand‐searched. A Preferred Reporting Items for Systematic Reviews and Meta‐Analyses (PRISMA) flow diagram (Fig. S1) summarised study selection. Included studies were tabulated with details on cohort size, follow‐up, use of validated PROMs, and URF closure outcomes.

## Results

### Clinical and Perioperative Characteristics

Baseline and clinical characteristics are depicted in Table 1. Between 2014 and 2024, 29 men underwent URF repair. The median (IQR) age at surgery was 68 (61–71) years, length of stay was 11 (9–14) days, and body mass index (BMI) was 26 (23–28) kg/m^2^. Hypertension (45%) was the most frequent comorbidity. Among the five patients (17%) who had a history of pelvic radiotherapy, one received it as primary therapy, three as adjuvant therapy, and one as salvage therapy.

**Table 1:** Clinical, URF, and surgical characteristics of 29 patients undergoing open URF repair after local prostate cancer treatment between January 2014 and June 2024.

Characteristic	Value
**Clinical baseline**	
Age, years, median (IQR)	68 (61–71)
BMI, kg/m^2^, median (IQR)	26 (23–28)
Smoking status, *n* (%)	
Never	19 (66)
Former	6 (21)
Current	4 (14)
Diabetes, *n* (%)	3 (10)
Hypertension, *n* (%)	13 (45)
Hyperlipidaemia, *n* (%)	8 (28)
Coronary artery disease, *n* (%)	6 (21)
American Society of Anesthesiologists physical status, *n* (%)	
I – A normal healthy patient	1 (3.5)
II – A patient with mild systemic disease	21 (72)
III – A patient with severe systemic disease	7 (24)
**Prostate cancer treatment**	
Time from radical prostatectomy to URF repair, months, median (IQR)	10 (4–13)
Radical prostatectomy approach, *n* (%)	
Open	18 (62)
Laparoscopic	2 (6.9)
Robot‐assisted	9 (31)
Prior pelvic or abdominal radiotherapy for prostate cancer, *n* (%)	5 (17)
Preoperative diagnostics, *n* (%)	
MRI	5 (18)
CT	7 (24)
Cystoscopy	11 (38)
Cystography	28 (97)
Rectoscopy	8 (28)
Foley catheter at admission, *n* (%)	21 (72)
Suprapubic catheter at admission, *n* (%)	5 (17)
Enterostomy at admission, *n* (%)	24 (83)
Symptomatology at presentation, *n* (%)	
Pneumaturia	7 (24)
Recurrent UTIs	6 (21)
Dysuria	6 (21)
Faecaluria	3 (10)
Rectal urine leakage	14 (48)
**URF**	
Prior URF repair/redo cases, *n* (%)	13 (45)
1 prior URF repair	9 (31)
2 prior URF repairs	1 (3.5)
3 prior URF repairs	2 (6.9)
4 prior URF repairs	1 (3.5)
Time from last repair to current redo URF repair, months, median (IQR)	5 (4–6)
**Operation**	
Operation approach, *n* (%)	
Transperineal	26 (90)
Transabdominal	3 (10)
Operating time, min, median (IQR)	90 (80–107)
Length of stay, days, median (IQR)	11 (9–14)

Percentages may not add up to 100%, as they are rounded.

Surgical history included open (62%), laparoscopic (6.9%), and robot‐assisted (31%) prostatectomy. The median (IQR) interval from prostatectomy to URF repair was 10 (4–13) months. In all, 13 men (45%) had undergone unsuccessful URF repair elsewhere.

Common presenting symptoms included rectal urine leakage (48%), pneumaturia (24%), recurrent UTIs (21%), and dysuria (21%). At surgery, 17% had a suprapubic catheter and 83% had an enterostomy. The median (IQR) operating time was 90 (80–107) min. A transperineal approach was used in 26 patients (90%), whereas a transabdominal approach was chosen in three (10%). Among the latter, one patient had a history of adjuvant pelvic radiotherapy and underwent concomitant left‐sided ureteroneocystostomy for distal ureteric stenosis. One patient had previously undergone three failed transperineal repairs and one fibrin glue closure attempt. The third patient had a history of salvage prostatectomy following primary radiotherapy and severe obesity (BMI 48 kg/m^2^). In these cases, a transabdominal approach was selected based on anticipated surgical complexity and the need for extensive exposure or additional reconstructive procedures.

One patient died in hospital from myocardial infarction, unrelated to the URF repair; this was the only major postoperative event (Clavien–Dindo Grade >III).

### Fistula Recurrence and Re‐intervention‐Free Survival

**Fig. 1:**
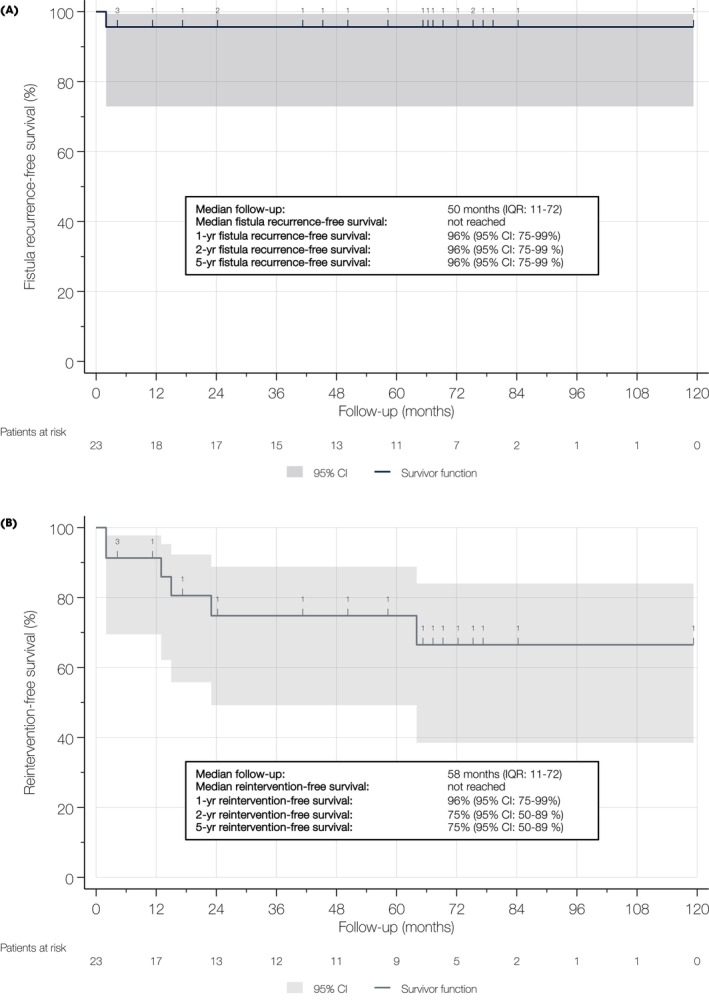
Kaplan–Meier curve depicting URF recurrence‐free **(A)** and re‐intervention‐free survival **(B)** in 23 patients undergoing open URF repair.

Overall, six patients (21%) were lost to follow‐up and excluded from survival analyses. At a median (IQR) follow‐up of 50 (11–72) months, one recurrence was documented, yielding a URF closure rate of 97% and an estimated URF recurrence‐free survival of 96% at 2 and 5 years (Fig. 1A).

The median (IQR) follow‐up for any re‐intervention for disease‐related events was 58 (11–72) months. Six patients (21%) required additional procedures (Fig. 1B), including artificial urinary sphincter implantation, penile prosthesis placement, cystoprostatectomy for recurrent cancer, transurethral resection for anastomotic stenosis, urinary diversion for iatrogenic bladder injury during self‐catheterisation, and redo URF repair. This corresponded to a re‐intervention‐free survival of 75% at 2 and 5 years. When grouped by indication, there was one (17%) re‐intervention for oncological reasons, four (67%) for disease‐related sequelae, and one (17%) for true URF recurrence.

### The PROMs

The PROMs assessment was completed at a median (IQR) of 71 (33–78) months with complete data from 17 patients (59%). Five patients (17%) had died during follow‐up. There was no statistically significant difference in clinical characteristics in PROMs responders vs non‐responders (Table S1), indicating a low likelihood of non‐response bias in our PROMs analyses.

**Fig. 2:**
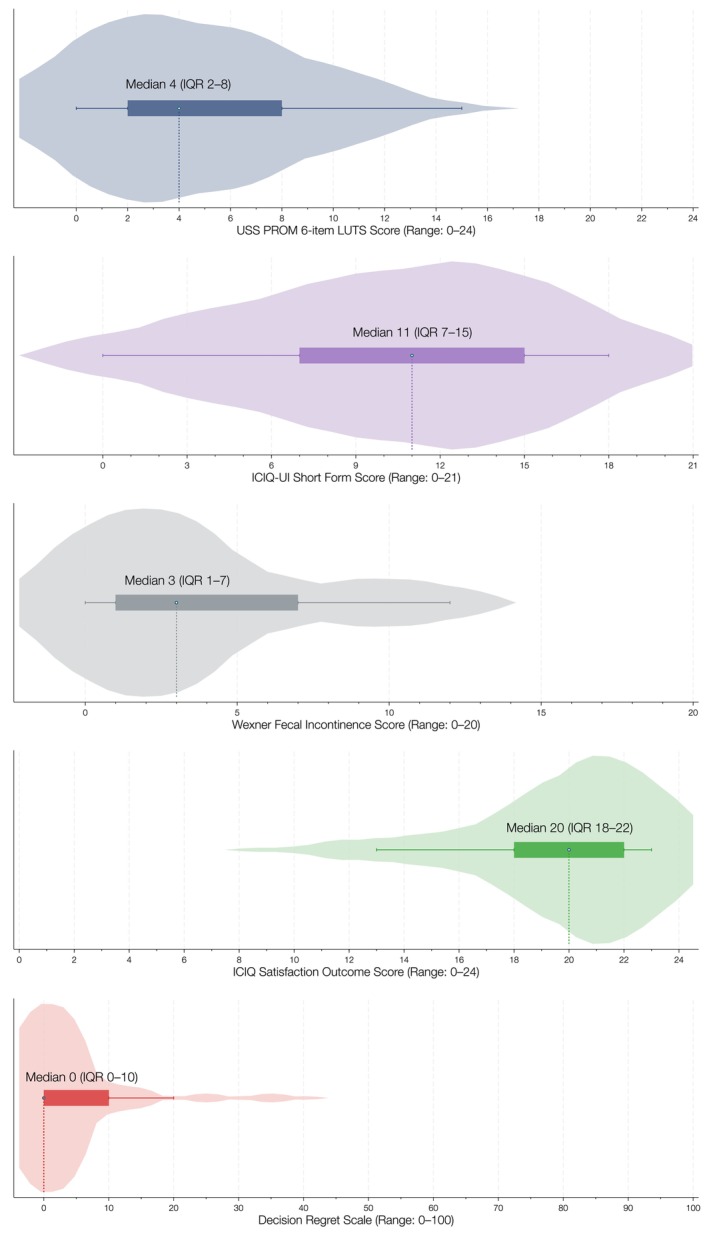
Violin plots illustrating the distribution of scores for validated PROMs in 17 of 29 patients undergoing open URF repair.

**Fig. 3:**
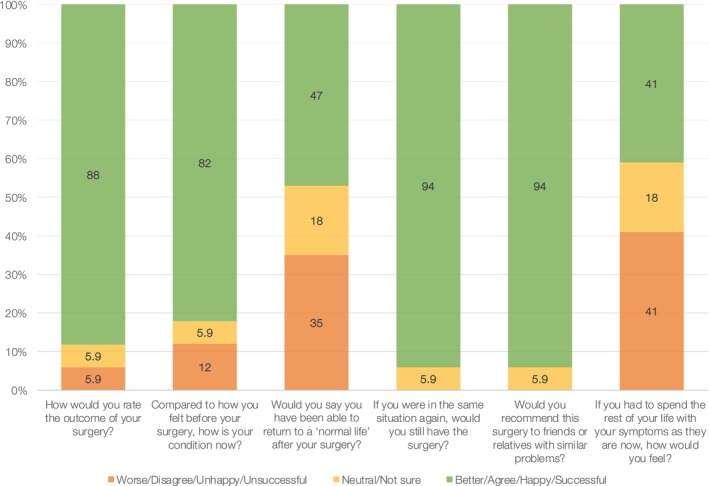
The ICIQ‐S outcomes questions survey results (*n* = 17). Percentages may not add up to 100%, as they are rounded.

The distribution of PROMs results is illustrated in Fig. 2. The median (IQR) postoperative LUTS score was 4 (2–8), indicating low symptom burden. The median (IQR) ICIQ‐UI SF score was 11 (6–15), consistent with moderate urinary incontinence [13]. The median (IQR) Wexner faecal incontinence score was 3 (1–9), reflecting minimal faecal incontinence. The median (IQR) ICIQ‐S outcome score was 21 (18–23) and global satisfaction was 9 (8–10), indicating high satisfaction. Figure 3 illustrates the distribution of responses to the six individual ICIQ‐S items, which collectively form the ICIQ‐S outcome score. The median (IQR) DRS score was 0 (0–10), indicating negligible decisional regret. The individual patient responses to the five items of the DRS are shown in Table S2.

### Scoping Review

Nine studies comprising 363 patients met the inclusion criteria [6, 12, 14, 15, 16, 17, 18, 19, 20]. Reported URF closure rates ranged from 73% to 100% (Table 2 [6, 12, 14, 15, 16, 17, 18, 19, 20]). Five studies included PROMs: four used validated questionnaires, primarily addressing urinary or faecal incontinence [16, 18, 19, 20].

**Table 2:** Publications on outcomes after URF repair as identified by a scoping literature review on 14 January 2025.

References	Year of publication	Patients, *n*	Surgical approach	Median/mean follow‐up, months	Success rate, %*	PROMs
Zmora et al. [14]	2003	11	Transperineal	18	73	NR
Lane et al. [15]	2006	22	York Mason Abdominoperineal	29	78	NR
Ghoniem et al. [16]	2008	25	Transperineal	28	96	UDI‐6, Wexner faecal incontinence score, VAS patient satisfaction/operation success, IPSS QoL item
Mundy and Andrich [17]	2011	40	Transabdominal Transperineal Abdominoperineal	12	100	NR
Rouanne et al. [18]	2011	10	Modified York Mason	24	100	Wexner faecal incontinence score
Pfalzgraf et al. [12]	2014	17	Transabdominal Transperineal Abdominoperineal	73	93	Non‐validated QoL questionnaire including questions regarding sexual and urinary function and treatment satisfaction
Harris et al. [6]	2017	201	Transperineal Abdominoperineal	6	93	NR
Theveniaud et al. [19]	2018	16	York Mason Transperineal	40	81	Wexner faecal incontinence score, ICSmale questionnaire, IIEF‐5
Sbizzera et al. [20]	2022	21	Transperineal	27	95	USP questionnaire, St Mark's incontinence score, POSAS

ICS, International Continence Society; IIEF‐5, five‐item version of the International Index of Erectile Function; NR, not reported; POSAS, Patient and Observer Scar Assessment Scale; QoL, quality of life; UDI‐6, six‐item version of the Urogenital Distress Inventory; USP, Urinary Symptom Profile; VAS, visual analogue scale.

* Defined as re‐intervention‐free survival.

## Discussion

Although indications for radical prostatectomy are becoming more selective, demographic trends suggest that absolute procedure numbers will remain high. Consequently, URFs will persist as a clinically significant complication [1]. This study integrates long‐term surgical outcomes with a comprehensive panel of validated PROMs in patients undergoing URF repair.

Our URF closure rate aligns with previously published series, reinforcing the durability of surgical repair across varying techniques [2], although long‐term data beyond 3 years are scarce in the literature. Interposition of vascularised tissue is considered fundamental to the long‐term success of fistula repair, in accordance with established surgical principles [21]. The high proportion of patients in our cohort with previous failed URF repairs highlights the importance of referral to specialised tertiary reconstructive centres. Despite 45% being redo cases, excellent outcomes were achieved, consistent with other high‐volume series [6, 17, 20].

Our study also provides important insights into patient‐centred outcomes. Five studies identified in our scoping review reported PROMs after URF repair, of which four used validated measures, primarily capturing urinary or faecal incontinence. Direct numerical comparisons with our findings are inappropriate due to differing instruments, scoring systems, and validation samples. However, qualitative comparison suggests that our cohort's degree of urinary incontinence – moderate according to an ICIQ‐UI SF classification [13] – is broadly comparable to previously reported six‐item version of the Urogenital Distress Inventory or Urinary Symptom Profile results [16, 20]. Faecal continence outcomes were favourable, with a median Wexner faecal incontinence score of 3, consistent with previous reports [16, 18, 19]. Nevertheless, variation in faecal outcomes likely reflects multiple contributing factors, including radiation‐induced tissue changes, local tissue quality, and baseline sphincter function.

Overall satisfaction was high, as reflected by the ICIQ‐S outcome score and the dedicated satisfaction‐with‐surgery item. Many patients regard URF repair as the only meaningful therapeutic option for a profoundly debilitating condition and therefore accept residual urinary and faecal symptoms in exchange for definitive URF closure. This is underscored by the individual item responses of the six ICIQ‐S questions (Fig. 3). When asked directly about the procedure and its outcome—specifically whether they would choose it again or recommend it to others with similar problems—the vast majority expressed positive views. In contrast, responses to the items assessing the ability to return to a normal life and how patients would feel if their current symptoms persisted lifelong were more heterogeneous and tended toward less favourable ratings. This pattern indicates that, although patients are satisfied with the procedure and accept its outcomes, the underlying condition continues to exert a substantial and enduring impact on daily functioning. This interpretation is supported by the 5‐year re‐intervention‐free survival of 75%, demonstrating that a meaningful subset of patients require additional procedures due to the complexity of the disease and the long‐term sequelae of prostate cancer treatment. Nevertheless, decisional regret regarding URF repair was negligible, reinforcing the procedure's role as a key salvage option despite the persistence of symptoms attributable to the primary condition. Importantly, all patients underwent thorough preoperative counselling regarding the available treatment options, including URF repair with a potentially complex and prolonged perioperative course vs permanent urinary diversion, which may be less invasive but potentially associated with inferior quality‐of‐life outcomes. We believe that this structured counselling and expectation management contributed to the negligible decisional regret observed, as patients were well informed and prepared for the anticipated outcomes of URF repair.

While our study offers valuable insights, several limitations must be acknowledged. The retrospective, single‐centre design and limited sample size constrain the generalisability of our findings and preclude multivariable risk‐factor analyses due to insufficient statistical power. Furthermore, the potential for non‐response bias exists, as patients who are more satisfied with their outcomes may be more inclined to complete follow‐up questionnaires. Nevertheless, this bias is likely limited, as patients experiencing dissatisfaction or unresolved concerns may also be motivated to report their experiences and there were no statistical differences in the re‐intervention rate when comparing PROMs responders and non‐responders.

Pre‐URF repair functional status regarding urinary and faecal continence could not be assessed in a standardised manner. Consequently, postoperative observations must be interpreted with caution, as a substantial portion of the remaining symptom burden is likely attributable to the underlying condition itself rather than the URF repair. Despite these limitations, our study provides a meaningful contribution to the current literature by offering detailed PROMs using a comprehensive set of validated instruments, including both guideline‐endorsed and innovative measures.

In summary, open URF repair achieves reliable and durable URF closure with favourable long‐term outcomes, even in complex or radiation‐associated cases. Most patients report high satisfaction and negligible decisional regret, reflecting both technical success and substantial relief from debilitating symptoms. Although moderate urinary incontinence persists in some patients – likely due to prior oncological treatments – voiding function and faecal continence are generally preserved. These findings support open URF repair as an effective strategy for restoring function and improving quality of life in this challenging patient population.

## Disclosure of Interests

All authors have no conflicts of interest to disclose.

## Supporting information


**Fig. S1.** Preferred Reporting Items for Systematic Reviews and Meta‐Analyses flow diagram illustrating the article selection process for the scoping review on outcomes after URF repair.

**Table S1.** Comparison of clinical characteristics between PROMs responders and non‐responders.

**Table S2.** Individual patient responses to the five items of the Decision Regret Scale in 17 of 29 patients undergoing URF repair.
