# Supplementary figures and images for: Long‐term patient‐reported outcomes of open urorectal fistula repair after prostate cancer treatment
Source: BJU Int. 2026 Mar 13;137(6):1067–75. doi: 10.1111/bju.70233 (PMC13168923; doi:10.1111/bju.70233)

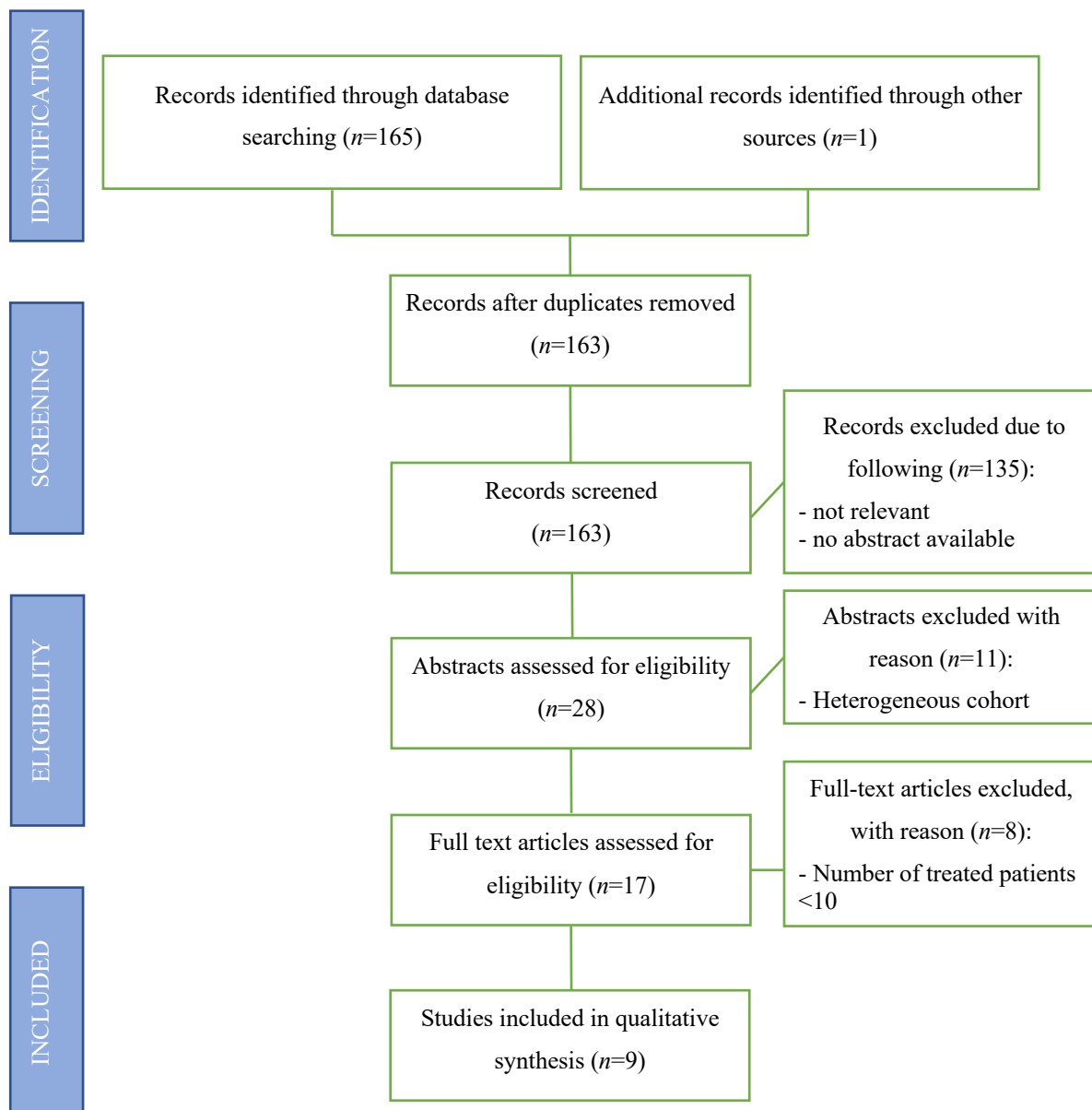

Supplement: Supplementary file 1 — Fig. S1. Preferred Reporting Items for Systematic Reviews and Meta‐Analyses flow diagram illustrating the article selection process for the scoping review on outcomes after URF repair. [file BJU-137-1067-s001.pdf]
