# Supplementary material for: Long‐term patient‐reported outcomes of open urorectal fistula repair after prostate cancer treatment
Source: BJU Int. 2026 Mar 13;137(6):1067–75. doi: 10.1111/bju.70233 (PMC13168923; doi:10.1111/bju.70233)
Supplement: Supplementary file 2 — Table S1. Comparison of clinical characteristics between PROMs responders and non‐responders. [file BJU-137-1067-s002.pdf]

Suppl. Table 1 – Comparison of clinical characteristics between PROM responders and non-responders.

	PROM responders *n*=17	PROM non-responders *n*=12	*p* value
Clinical baseline characteristics			
Age (yr), median (IQR)	67 (60–71)	69 (66–74)	0.3
BMI, median (IQR)	27 (23–28)	25 (22–29)	0.6
Smoking status, *n* (%)			0.7
Never	11 (65)	8 (67)	
Former	3 (18)	3 (18)	
Current	3 (18)	1 (8.3%)	
Prostate cancer treatment characteristics			
Time from radical prostatectomy to URF repair (mo), median (IQR)	10 (4–12)	10 (6–17)	0.3
Radical prostatectomy approach, *n* (%)			0.3
Open	9 (53)	9 (75)	
Laparoscopic	2 (12)	0 (–)	
Robot-assisted	6 (35)	3 (25)	
Prior pelvic or abdominal radiotherapy for prostate cancer, *n* (%)	15 (88)	9 (75)	0.4
URF characteristics			
Prior URF repair/redo cases, *n* (%)	9 (53)	4 (33)	0.3
Operative characteristics			
Operative approach, *n* (%)			0.8
Transperineal	15 (88)	11 (92)	
Transabdominal	2 (12)	1 (8.3)	
Outcomes			
Reintervention, *n* (%)	4 (24)	2 (17)	>0.9

BMI = body mass index; IQR = interquartile range; PROM = patient-reported outcome measure; URF = urorectal fistula.
