# Supplementary material for: Long‐term patient‐reported outcomes of open urorectal fistula repair after prostate cancer treatment
Source: BJU Int. 2026 Mar 13;137(6):1067–75. doi: 10.1111/bju.70233 (PMC13168923; doi:10.1111/bju.70233)
Supplement: Supplementary file 3 — Table S2. Individual patient responses to the five items of the Decision Regret Scale in 17 of 29 patients undergoing URF repair. [file BJU-137-1067-s003.pdf]

Suppl. Table 2 – Individual patient responses to the five items of the Decision Regret Scale in 17 of 29 patients undergoing urorectal fistula repair.

**Decision Regret Scale items; *n* (%)**	Strongly agree	Agree	Neither agree nor disagree	Disagree	Strongly disagree
1. It was the right decision.	15 (88)	2 (12)	0 (–)	0 (–)	0 (–)
2. I regret the choice that was made.	0 (–)	0 (–)	1 (5.9)	2 (12)	14 (82)
3. I would go for the same choice if I had to do it over again.	15 (88)	1 (5.9)	1 (5.9)	0 (–)	0 (–)
4. The choice did me a lot of harm.	0 (–)	2 (12)	1 (5.9)	2 (12)	12 (71)
5. The decision was a wise one.	13 (76)	4 (24)	0 (–)	0 (–)	0 (–)
